# Scales on Quality of Life in patients with spinal cord injury: integrative review

**DOI:** 10.1590/S1679-45082014RW2591

**Published:** 2014

**Authors:** Rita Lacerda Aquarone, Ana Cristina Mancussi e Faro

**Affiliations:** 1 Hospital Israelita Albert Einstein, São Paulo, SP, Brazil; 2 Universidade de São Paulo, São Paulo, SP, Brazil

**Keywords:** Spinal cord injury, Quality of life, Questionnaires

## Abstract

Studies evaluating the Quality of Life of individuals with spinal cord injury using different research tools demonstrate that the Quality of Life scores are considered low both in national and international studies. The objective of this review was to characterize the international scientific production about the most used scales to assess Quality of Life in patients with spinal cord injury. We examined articles on Quality of Life of patients with spinal cord injury published over the last 5 years and indexed in the National Library of Medicine (PUBMED). During this period, 28 articles met the inclusion criteria. Eleven studies were conducted in the United States, five articles were published in Australia, and four in Canada. Brazil, France, Holland, India, Japan, Norway, Singapore and Switzerland contributed with one study each. The scientific articles were published in 13 high impact factor journals. Seven different instruments to assess Quality of Life were used in the studies: Satisfaction with Life Scale, Short Form (36) Health Survey, The Brief Version of the WHO Quality of Life Questionnaire (WHOQOL-BREF), Comprehensive Quality of Life Scale, Life Situation Questionnaire-Revised, Quality of Well-Being Scale and the SF-12^®^ Health Survey. The articles examined underscore the impact of spinal cord injury in the Quality of Life of patients, demonstrating how this condition impairs their lives, mainly socially, but followed by the physical aspects. Despite the studies have different goals they all acknowledge that further studies are necessary in order to determine the Quality of Life of patients with spinal cord injury. Specific instruments should be chosen or developed and validated in order to fulfill this purpose

## RESUMO

Estudos sobre avaliação de escalas de Qualidade de Vida em indivíduos com lesão medular, mesmo com diferentes ferramentas de pesquisa, mostram que, tanto em estudos nacionais como em outros países, os escores de avaliação são considerados baixos. O objetivo desta revisão foi caracterizar na produção científica internacional o uso das escalas para avaliação da Qualidade de Vida mais usadas em pacientes com lesão medular. Foram analisados artigos que versavam sobre estudos relacionados à Qualidade de Vida em pacientes com lesão medular, nas publicações dos últimos 5 anos, indexadas na *National Library of Medicine* (PUBMED). No período analisado, 28 artigos atenderam aos critérios de inclusão. Foram realizadas 11 pesquisas nos Estados Unidos, 5 artigos foram publicados na Austrália, 4 no Canadá, Brasil, França, Holanda, Índia, Japão, Noruega, Singapura e Suíça tiveram 1 trabalho cada. Foram publicados em 13 revistas de impactos. Foram usadas sete escalas diferentes de Qualidade de Vida: *Satisfaction with Life Scale, Short Form (36) Health Survey*, Avaliação de Qualidade de Vida da Organização Mundial da Saúde, *Comprehensive Quality of Life Scale, Life Situation Questionnaire-Revised, Quality of Well-Being Scale* e SF-12^®^ *Health Survey*. Os artigos analisados destacaram o impacto da lesão medular na Qualidade de Vida dos pacientes, demonstrando como essa condição acarreta comprometimento, principalmente dos aspectos sociais, seguidos dos aspectos físicos. Mesmo com objetivos diferentes, reconhece-se que são necessários mais estudos para determinação da Qualidade de Vida em pacientes com lesão medular, devendo, ainda, serem escolhidos ou construídos e validados instrumentos específicos para esse fim.

## INTRODUCTION

The expression “Quality of Life” (QL) was first used by the president of the United States, Lyndon Johnson, in 1964, when he stated that “these goals cannot be measured by the size of our bank balances. They can only be measured in the quality of the lives that our people lead”. At first the interest in concepts such as “standard of living” and “Quality of Life” was shared by social scientists, philosophers and politicians. The negative impact of technology advances in Medicine and Life Sciences was its progressive dehumanization. Thus, human and biological sciences were concerned with the definition of “Quality of Life”. It should value parameters that go beyond controlling symptoms, reducing mortality or increasing life expectancy.^(1)^

The World Health Organization (WHO) defines QL as: “individuals' perception of their position in life in the context of the culture and value systems in which they live and in relation to their goals, expectations, standards and concerns”. This definition implies the idea that the concept of QL is subjective, multidimensional, and includes both positive and negative elements.^(1)^

Studies that assessed QL of individuals with spinal cord injury (SCI), despite having used different research tools, demonstrate that quality of life scores are considered low both national and international studies.^(2)^

Quality of life assessment is important because it broadens the decisions made by the health team, extending them to healthcare programs and policies. Many researchers are unanimous in stating that the failure of many programs lie on the fact that they are based on the perception of health professionals, with interventions that not connected to the social and QL conditions. Consequently, there are not many studies and the reals needs, beliefs and motivations of patients are undervalued.^(3)^

One of the essential aspects in assessing QL is to determine what is important to the individual, especially when the instrument is used in different cultures.

## OBJECTIVE

The objective of this study was to characterize the international scientific production on quality of life scales used to assess the quality of life of patients with spinal cord injury.

## METHODS

The following steps were followed in preparing this integrative review: establishing the hypothesis and the objectives of the integrative review; determining the criteria to include and to exclude articles (sample selection); determining the information to be extracted from the scientific papers selected; result analysis; results discussion; and the last step was to produce this review.^(4)^

The guiding question chosen for this study was: “Which are the scales used to assess the QL of patients with spinal cord injury?”

The search for articles published in the international literature was carried out using an online database. This study included scientific papers about studies that examined the QL of patients with spinal cord injury and that were published over the last 5 years (2007-2012) and indexed by the National Library of Medicine (PUBMED).

The following terms were used in our search: “spinal cord injury” AND “quality of life”.

In order to refine the sample, the following inclusion criteria were used: articles published in English, indexed by PUBMED, published over the last 5 years, full text available online; studies with adult human beings focused on QL of patients with SCI.

Exclusion criteria – studies that do not report results, such as editorials, letters to the editor and review articles; studies that failed to use a Quality of Life scale; or those that presented duplicity of sources of information used were excluded.

Research subfields were not established in PUBMED. By applying the following search limits: free full text, Humans, English, All Adult: 19+ years, published in the last 5 years, 75 articles were identified and after analysis, 28 articles met the inclusion criteria previously established.

The articles found were firstly examined according to their title and abstract. Twenty-eight articles met the inclusion criteria, and we read them from beginning to end. In order to categorize the papers, information from them was organized in a spreadsheet, using a validated data collection tool.^(5)^ The following variables were examined: year of publication, article title, city/country, study topic, objectives and scales used.

Content was then examined according to the objective of each study. After the reading step, the articles and the tools were organized and catalogued for future searches.

Results and data discussion are described next with the support of graphs and tables, allowing the reader to apply the results of this integrative review and to replicate the methodological steps.

## RESULTS

Twenty-eight articles that met the inclusion criteria were examined in this integrative review. Out of the articles included, 11 studies were carried out in the USA, 5 articles were from Australia and 4 from Canada. Brazil, France, Holland, India, Japan, Norway, Singapore and Switzerland had one article included.

As for year of publication, two articles were published in 2012 (7.2%); ten in 2011 (35.7%); one (3.5%) in 2010; six in 2009 (21.5%); eight in 2008 (28.6%), and one (3.5%) in 2007.

**Figure 1:**
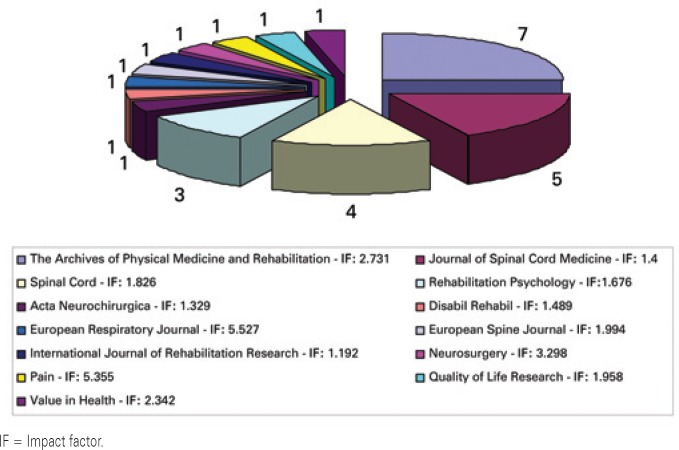
Distribution of articles according to journal and impact factor

The articles that met the inclusion criteria were published in 13 high impact journals (Figure 1).

All articles used a Quality of Life assessment scale in patients with SCI. Seven different scales were used, namely Satisfaction with Life Scale (SWLS, 9 articles), Short Form (36) Health Survey (SF-36, 8 articles), The Brief WHO Quality of Life Questionnaire (WHOQOL-bref, 7 articles), Comprehensive Quality of Life Scale (ComQol, 1 article), Life Situation Questionnaire-Revised (LSQ-R, 1 article), Quality of Well-Being Scale (QWB-SA, 1 article), and SF-12^®^ Health Survey (1 article).

The articles had different objectives, populations and results. Only one article had an exclusive male population; all the others, *i.e.*, 27 articles described studies that included both men and women with different level and severity of the SCI.

**Figure 2:**
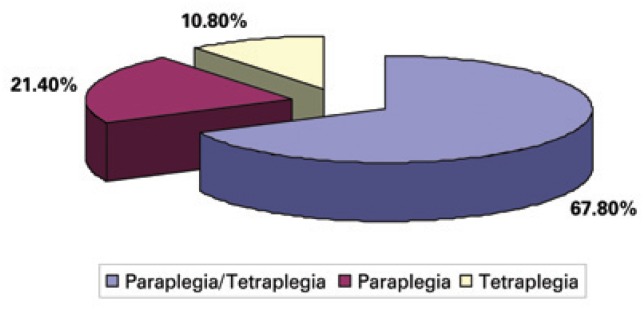
Distribution of scientific papers according to diagnosis and population studied

Depending on the level of the SCI, the patient may be diagnosed with either paraplegia or quadriplegia. The articles examined included both populations or focused on just one of them – the population of three articles had quadriplegia, six other articles focused on individuals with paraplegia and 19 articles included both paraplegic and quadriplegic subjects (Figure 2).

Methodology used in the studies: 8 were cross-sectional (28.5%); 8 were retrospective (28.5%); 7 were prospective (25%); 2 (7.1%) were longitudinal; 2 were observational (7.1%); and 1 was a randomized study (3.8%).

Chart 1 presents a summary of the studies conducted and discussed, according to the year of publication.

**Chart 1:** Article identification and analysis according to the study subject, authors, scale used and journal

Authors	Country/year	Title	Journal	Scale
Wijesuriya et al.	Australia/2012	Impact of fatigue on the health-related quality of life in persons with spinal cord injury	The Archives of Physical Medicine and Rehabilitation	WHOQOL-bref
Pershouse et al.	Australia/2012	Investigating changes in quality of life and function along the lifespan for people with spinal cord injury	The Archives of Physical Medicine and Rehabilitation	WHOQOL-bref
Migliorini et al.	Australia/2011	Quality of life in adults with spinal cord injury living in the community	Spinal Cord	ComQol
Kumar et al.	Canada/2011	Spinal cord stimulation is effective in management of complex regional pain syndrome I: fact or fiction	Neurosurgery	SF-36
Hetz et al.	Canada/2011	Secondary complications and subjective well-being in individuals with chronic spinal cord injury: associations with self-reported adiposity	Spinal Cord	SWLS
Teo et al.	Cingapore/2011	Health of people with spinal cord injury in Singapore: implications for rehabilitation planning and implementation	Disabil Rehabil	SWLS
Gautschi et al.	Switzerland/2011	Health-related quality of life following spinal cordectomy for syringomyelia	Acta Neurochirurgica	SF-36
Kemp et al.	USA/2011	Effects of reduction in shoulder pain on quality of life and community activities among people living long-term with SCI paraplegia: a randomized control trial	Journal of Spinal Cord Medicine	WHOQOL-bref
Riggins et al.	USA/2011	The relationship between quality of life and change in mobility 1 year postinjury in individuals with spinal cord injury	The Archives of Physical Medicine and Rehabilitation	WHOQOL-bref
Vogel et al.	USA/2011	Long-term outcomes of adults with pediatric-onset spinal cord injuries as a function of neurological impairment	Journal of Spinal Cord Medicine	SF-12
Charlifue et al.	USA/2011	Mechanical ventilation, health, and quality of life following spinal cord injury	The Archives of Physical Medicine and Rehabilitation	SWLS
Myaskovsky et al.	USA/2011	The association of race, cultural factors, and health-related quality of life in persons with spinal cord injury	The Archives of Physical Medicine and Rehabilitation	SWLS
Kortte et al.	USA/2010	Positive psychological variables in the prediction of life satisfaction after spinal cord injury	Rehabilitation Psychology	SWLS
Blanes et al.	Brazil/2009	Quality of life and self-esteem of persons with paraplegia living in São Paulo, Brazil	Quality of Life Research	SF-36
McVeigh et al.	Canada/2009	Influence of sport participation on community integration and quality of life: a comparison between sport participants and non-sport participants with spinal cord injury	Journal of Spinal Cord Medicine	WHOQOL-bref
Adler et al.	France/2009	Diaphragm pacing restores olfaction in tetraplegia	European Respiratory Journal	SWLS
Kotani et al.	Japan/2009	Minimum 2-year outcome of cervical laminoplasty with deep extensor muscle-preserving approach: impact on cervical spine function and quality of life	European Spine Journal	WHOQOL-bref
Krause et al.	USA/2009	Life satisfaction and self-reported problems after spinal cord injury: measurement of underlying dimensions	Rehabilitation Psychology	LSQ-R
deRoon-Cassini et al.	USA/2009	Psychological well-being after spinal cord injury: perception of loss and meaning making	Rehabilitation Psychology	SF-36
Lee et al.	Austrália/2008	Validity, responsiveness, and minimal important difference for the SF-6D health utility scale in a spinal cord injured population	Value in Health	SF-36
Tonack et al.	Canadá/2008	Predicting life satisfaction after spinal cord injury in a Canadian sample	Spinal Cord	SWLS
Vranken et al.	Holanda/2008	Pregabalin in patients with central neuropathic pain: a randomized, double-blind, placebo-controlled trial of a flexible-dose regimen	Pain	SF-36
Singh et al.	Índia/2008	Quality of life of people with spinal cord injury in Northern India	International Journal of Rehabilitation Research	WHOQOL-bref
Lidal et al.	Noruega/2008	Health-related quality of life in persons with long-standing spinal cord injury	Spinal Cord	SF-36
Chen et al.	USA/2008	Change in life satisfaction of adults with pediatric-onset spinal cord injury	The Archives of Physical Medicine and Rehabilitation	SWLS
Stevens et al.	Estados Unidos/2008	Physical activity and quality of life in adults with spinal cord injury	Journal of Spinal Cord Medicine	Quality of Well-Being Scale
Anderson et al.	Estados Unidos/2008	Coping with spinal cord injury: strategies used by adults who sustained their injuries as children or adolescents	Journal of Spinal Cord Medicine	SWLS
Middleton et al.	Estados Unidos/2007	Relationship between quality of life and self-efficacy in persons with spinal cord injuries	The Archives of Physical Medicine and Rehabilitation	SF-36

Source: PUBMED.

WHOQOL-bref: World Health Organization Quality of Life Assessment; ComQol: Comprehensive Quality of Life Scale; SF-36: Short Form (36) Health Survey; SWLS: Satisfaction with Life Scale; SF-12: SF-12^®^ Health Survey; LSQ-R: Life Situation Questionnaire-Revised; SCI: spinal cord injury.

## DISCUSSION

Results indicate that in 2011 there was an increase in the number of publications about QL in patients with SCI, with variations in previous and subsequent years.

The studies objectives and foci were different, but except for one study with war veterans with SCI, all the others included men and women in their QL investigation. The studies also combined patients with paraplegia and quadriplegia because QL should not be better or worse because of the physical status.

Quality of life is complex and perhaps this is why it is no surprise that it has no consensus definition or standard measurement tool. It does not mean that there was a shortage of ideas, there are more than one hundred QL measurement tools, but each one of them contain an idiosyncratic mix of dependent variables.^(6)^ It is also noteworthy that many QL measurement tools have been developed to highly selected groups – particularly regarding the scales developed to monitor medical conditions or procedures, and this is why they should not be applied in the general population. However, even more general tools should not be used in the entire population. Measurement tools developed to the general adult population should not be used in subgroups such as individuals with cognitive impairment and children. This is an important limitation because this means that the QL for these groups cannot be used as a standard to the general population.^(6)^

The SWLS is the most mentioned scale. It was developed to meet the need of a multi-item scale. It measures the level of satisfaction with life based on a cognitive process. The SWLS psychometric properties seem to be favorable in American populations. Later, it was concluded that this scale could be used in different age groups. This self report tool has five items, the SWLS was originally developed as a 7-point Likert scale, in which respondents indicate how much they agree or disagree with each item.^(7)^

The SF-36 questionnaire has been broadly used because it is a generic, easy to administer and easy to understand questionnaire. Relatively short, it usually takes between 5 and 10 minutes to be administered. This tool came out from a health assessment questionnaire that contained 149 items, which was developed and tested in more than 22,000 patients as part of a health assessment study, The Medical Outcomes Study (MOS). The final version is a 36-item questionnaire divided in 8 subscales or domains, namely Physical Functioning (10 items), Role Limitations due to Physical Problems (4 items), Bodily Pain (2 items) General Health Perceptions (5 items), Vitality (4 items), Social Functioning (2 items), Role Limitations due to Emotional Problems (3 items), General Mental Health (5 items) and one more question comparing the current health status to the status one year before. This tool assesses both positive health aspects (well-being) as well as negative aspects (disease).^(8)^

One key element in QL assessment is to determine what is important to the individual, especially when the tool is used in different cultures. In this regard, the analysis carried out by the WHO Quality of Life group demonstrated that it is possible to develop a validated cross-cultural QL measurement tool by organizing a collaborative project in 15 centers. The outcome of this project was the development of the World Health Organization Quality of Life-100 (WHOQOL-100).^(9)^

Because of the need of short and rapidly administered tools, a shorter version of the WHOQOL-100 questionnaire was developed, the WHOQOL-bref instrument with 26 questions in its final version. The first question is about the overall quality of life and the second is about satisfaction with their health. The other 24 questions measure the following domains: physical, psychological, social relationships, and the environment. It can be used both in healthy as well as in individuals with chronic diseases and conditions. Besides being cross-cultural, the WHOQOL instruments value the individual perception, allowing for assessing QL in different groups and situations.^(10)^

The ComQol scale encompasses a contemporary understanding of QL. It was designed to be a self-administered. This scale was developed to be appropriate to any subgroup, *i.e.*, the adult population in general, individuals with intellectual disability or any cognitive impairment, 11 to 18-year old adolescents attending school. ComQol has seven domains: material well-being, health, productivity, intimacy, safety, community and emotional well-being.^(11)^

The LSQ-R is a subjective well-being scale that assesses an individual's current level of satisfaction in several life domains. It also requires respondents to examine life problems. The LSQ-R is embedded into a larger survey, the Life Situation Questionnaire (LSQ), which measures a broad range of SCI outcomes, including those related to employment, medical treatments, social activities and subjective well-being.^(12)^

The QWB-SA tool is a simple and general questionnaire with two main components. The first component is a descriptive system that determines health-related quality of life, HR-QoL, in 5 dimensions (mobility, self care, usual activities, pain/discomfort and anxiety/depression, each with three response options (1: no problems; 2: moderate problems; 3: severe problems). The second component is the Visual Analogue Scale (VAS), a 100-point scale in which zero means no pain and 100 means the worst imaginable pain.^(13)^

The SF-12^®^ Health Survey consists of 12 items divided in two components: mental and physical. This instrument is the shorter version of the SF-36. Zero indicates the worst, and 100 the best score.^(1)^

Based on the analysis of articles that used different measurement scales to assess the QL in individuals with SCI, it was possible to identify that QL encompasses psychological and social well-being, emotional functioning, health status, functional performance, satisfaction with life, social support and living standard. Adequate instruments should be used to assess the perception about specific conditions or patient's treatment conditions. This assessment is important because it allows for sharing outcomes from different parts of the world, and consequently, comparing quality of life in different cultures and social groups, as well as the efficiency of different treatment techniques.^(14)^

## CONCLUSION

The studies examined highlight the large variety of measurement scales used in the literature to assess the Quality of Life of patients with spine cord injury. They demonstrate that there is no specific scale for this population. Further studies should be examined or conducted, as well as specific instruments for this purpose.
